# Protein Expression Profile of HT-29 Human Colon Cancer Cells after Treatment with a Cytotoxic Daunorubicin-GnRH-III Derivative Bioconjugate

**DOI:** 10.1371/journal.pone.0094041

**Published:** 2014-04-09

**Authors:** Verena Natalie Schreier, Lilla Pethő, Erika Orbán, Andreas Marquardt, Brindusa Alina Petre, Gábor Mező, Marilena Manea

**Affiliations:** 1 Department of Chemistry, University of Konstanz, Konstanz, Germany; 2 MTA-ELTE Research Group of Peptide Chemistry, Budapest, Hungary; 3 Proteomics Facility, University of Konstanz, Konstanz, Germany; 4 Faculty of Chemistry, “Al. I. Cuza” University of Iasi, Iasi, Romania; 5 Zukunftskolleg, University of Konstanz, Konstanz, Germany; Ospedale Pediatrico Bambino Gesù, Italy

## Abstract

Targeted delivery of chemotherapeutic agents is a new approach for the treatment of cancer, which provides increased selectivity and decreased systemic toxicity. We have recently developed a promising drug delivery system, in which the anticancer drug daunorubicin (Dau) was attached *via* oxime bond to a gonadotropin-releasing hormone-III (GnRH-III) derivative used as a targeting moiety (Glp-His-Trp-Lys(Ac)-His-Asp-Trp-Lys(Dau = Aoa)-Pro-Gly-NH_2_; Glp = pyroglutamic acid, Ac = acetyl; Aoa = aminooxyacetyl). This bioconjugate exerted *in vitro* cytostatic/cytotoxic effect on human breast, prostate and colon cancer cells, as well as significant *in vivo* tumor growth inhibitory effect on colon carcinoma bearing mice. In our previous studies, H-Lys(Dau = Aoa)-OH was identified as the smallest metabolite produced in the presence of rat liver lysosomal homogenate, which was able to bind to DNA *in vitro*. To get a deeper insight into the mechanism of action of the bioconjugate, changes in the protein expression profile of HT-29 human colon cancer cells after treatment with the bioconjugate or free daunorubicin were investigated by mass spectrometry-based proteomics. Our results indicate that several metabolism-related proteins, molecular chaperons and proteins involved in signaling are differently expressed after targeted chemotherapeutic treatment, leading to the conclusion that the bioconjugate exerts its cytotoxic action by interfering with multiple intracellular processes.

## Introduction

Receptor-mediated drug delivery is a promising approach for the treatment of cancer, which may provide increased selectivity and decreased systemic toxicity compared to classical chemotherapy (i.e., administration of free anticancer drugs) [1]–[3]. Considering that receptors for certain regulatory peptides, such as gonadotropin-releasing hormone (GnRH; also known as luteinizing hormone-releasing hormone, LHRH), are highly expressed on a variety of cancer cells with relatively limited expression in normal tissues, they represent important molecular targets in cancer therapy [4]. Thus, GnRH derivative peptides could be employed as targeting moieties for the attachment and subsequent specific delivery of chemotherapeutic agents to GnRH-receptor (GnRH-R) positive cancer cells. After their internalization by receptor-mediated endocytosis, the bioconjugates are generally processed in lysosomes, leading to the release of the free drug or to the formation of drug-containing metabolites [5], [6].

**Figure 1:**
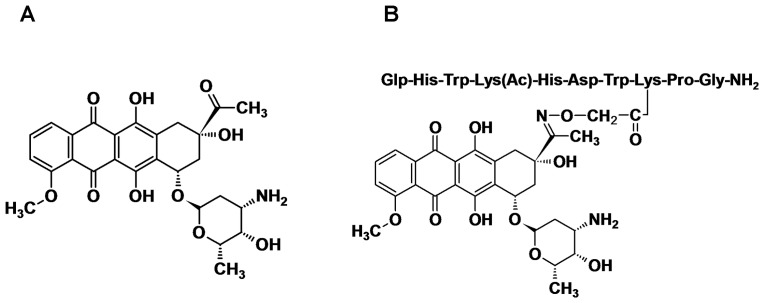
Structure representation of cytotoxic agents. (A) daunorubicin and (B) oxime bond-linked daunorubicin-GnRH-III derivative bioconjugate, GnRH-III[^4^Lys(Ac), ^8^Lys(Dau = Aoa)].

A promising native GnRH analog to be used as a targeting moiety is lamprey GnRH-III (Glp-His-Trp-Ser-His-Asp-Trp-Lys-Pro-Gly-NH_2_), which binds to GnRH-Rs, has an insignificant endocrine effect in mammals and exerts a direct antiproliferative effect on both hormone-dependent and -independent cancer cells [7]–[9]. In our previous work, various anthracycline-GnRH-III derivative bioconjugates have been designed, synthesized and biochemically characterized [10]–[12]. One of the most promising drug delivery systems developed to date in our laboratories consists of the anticancer drug daunorubicin (Dau) attached *via* an oxime bond to a GnRH-III derivative in which Ser in position 4 was replaced by Lys(Ac) [13]. Daunorubicin (Figure 1A) is a chemotherapeutic agent which interferes with the cell proliferation and division by mechanisms such as DNA intercalation, inhibition of topoisomerase II, free radical formation, lipid peroxidation, etc. Despite its clinical benefits, the administration of free Dau is followed by toxic side effects, the most severe one being cardiotoxicity [14], [15]. Therefore, the attachment of Dau to GnRH-based targeting moieties should provide increased selectivity and decreased systemic toxicity [12]. We have recently shown that the bioconjugate GnRH-III[^4^Lys(Ac), ^8^Lys(Dau = Aoa)] (Figure 1B) exerted *in vitro* cytostatic/cytotoxic effect on human breast, prostate and colon cancer cells, with IC_50_ values in low μM range. It is important to mention that on HT-29 colon cancer cells, the bioconjugate exerted higher cytostatic effect (IC_50_ = 7.4±2.6 μM) than the parent bioconjugate in which Dau was attached to the native peptide hormone (IC_50_ = 27.8±4.2 μM). Moreover, on colon carcinoma bearing mice, GnRH-III[^4^Lys(Ac), ^8^Lys(Dau = Aoa)] exerted significant *in vivo* tumor growth inhibitory effect (49.3% tumor growth inhibition relative to the untreated control group) [13]. Furthermore, H-Lys(Dau = Aoa)-OH was identified as the smallest drug-containing metabolite produced in the presence of rat liver lysosomal homogenate, which was able to bind to DNA *in vitro* [10], [13], result that could contribute to the understanding of the cytotoxic effect of the bioconjugate.

In order to get a deeper insight into the mechanism of action of GnRH-III[^4^Lys(Ac), ^8^Lys(Dau = Aoa)] bioconjugate, changes in the protein expression profile of HT-29 human colon cancer cells after treatment with the bioconjugate or free Dau were investigated by mass spectrometry-based proteomics. Our results indicate that several metabolism-related proteins, molecular chaperons and proteins involved in signaling are differently expressed after targeted chemotherapeutic treatment, leading to the conclusion that the bioconjugate exerts its cytotoxic action by interfering with multiple intracellular processes.

## Materials and Methods

### Materials

RPMI-1640 medium, fetal calf serum (FCS) and 3-(4,5-dimethylthiazol-2-yl)-2,5-diphenyltetrazolium bromide (MTT) were obtained from Sigma-Aldrich (Budapest, Hungary). Nonlinear immobilized pH gradient strips (IPG strips, pH 3–10 NL, 17 cm) were purchased from Bio-Rad Laboratories (Munich, Germany), while the bicinchoninic acid (BCA) assay kit was a Pierce product (Rockford, USA). Lysis buffer (0.15 M NaCl, 5 mM EDTA, 1% Triton-X100, 10 mM Tris-HCl pH = 7.4, 5 mM DTT and protease inhibitors), rehydration buffer (7 M urea, 2 M thiourea, 4% CHAPS, 40 mM Tris, 2% Servalyt 3–10), equilibration buffer (6 M urea, 2% SDS, 30% glycerol, 2.5 mM Tris-HCl) and running buffer (25 mM Tris, 192 mM Glycine and 0.1% SDS) were prepared in our laboratories.

### Synthesis and Chemical Characterization of GnRH-III[^4^Lys(Ac), ^8^Lys(Dau = Aoa)] Bioconjugate

The bioconjugate GnRH-III[^4^Lys(Ac), ^8^Lys(Dau = Aoa)] was prepared by a combination of solid phase peptide synthesis and chemoselective ligation in solution as previously reported [13]. Briefly, the aminooxyacetylated derivative of GnRH-III (Glp-His-Trp-Lys(Ac)-His-Asp-Trp-Lys(Aoa)-Pro-Gly-NH_2_) was synthesized on a Rink Amide MBHA resin according to Fmoc/tBu strategy, using an orthogonal protecting scheme for the lysine residues in positions 4 and 8 (Protocol S1). Daunorubicin was attached *via* oxime bond to the aminooxyacetylated GnRH-III derivative, reaction which was carried out in solution (0.2 M sodium acetate, pH 5) (Figure S1). After its purification by semipreparative HPLC, the bioconjugate (Glp-His-Trp-Lys(Ac)-His-Asp-Trp-Lys(Dau = Aoa)-Pro-Gly-NH_2_) was characterized by analytical HPLC and mass spectrometry (Protocol S2 and S3 and Figure S2).

### Cells

HT-29 (ATCC:HTB-38) human colon carcinoma cells were maintained in RPMI-1640 medium containing 10% FCS, L-glutamine (2 mM) and gentamicine (160 μg/mL). The cell cultures were maintained at 37°C in a humidified atmosphere with 5% CO_2_.

### *In vitro* Cytotoxic Effect

The *in vitro* cytotoxic effect of GnRH-III[^4^Lys(Ac), ^8^Lys(Dau = Aoa)] bioconjugate and free Dau was determined by MTT-assay. A number of 3×10^3^ cells per well were plated on 96-well plates. After 24 h incubation at 37°C, cells were treated for 6, 24, 48 and 72 h with the bioconjugate or free Dau dissolved in serum-free medium (concentration range: 2.6×10^−4^–10^2^ μM). Cells treated with serum-free medium for the same periods of time were used as a control. After that, the MTT solution was added to each well. After 3.5 h of incubation, purple crystals were formed by mitochondrial dehydrogenase enzyme of living cells. Cells were centrifuged for 5 min at 1000 g and the supernatant was removed. Crystals were dissolved in dimethyl sulfoxide and the optical density (OD) was measured at λ = 540 and 620 nm using an ELISA Reader (Labsystems MS reader, Finland). OD_620_ was subtracted from OD_540_ and the percent of cytotoxicity was calculated using the following equation:
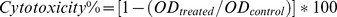
where OD_treated_ and OD_control_ corresponded to the optical densities of treated and control cells, respectively. Cytotoxicity % was plotted as a function of concentration, fitted to a sigmoidal curve and the IC_50_ value was determined on the basis of this curve. IC_50_ represented the concentration of bioconjugate or Dau required to achieve 50% inhibition *in vitro*.

### Preparation of Cell Lysates

In order to prepare the cell lysates, 5×10^5^ HT-29 human colon cancer cells per well were plated on 6-well plates. After 24 h incubation at 37°C, the cells were treated for 72 h with the bioconjugate (at a concentration of 3 μM) or free Dau (at a concentration of 0.15 μM). Cells treated with cell culture medium for the same period of time were used as a control. After incubation, the cells were centrifuged for 5 min at 1000 rpm, washed with phosphate buffered saline, pH = 7.3 and then a volume of 300 μL lysis buffer was added to each well. Samples were incubated for 30 min on ice and then the isolated protein mixtures were centrifuged at 16000 g for 20 min at 4°C. The protein content of the soluble fraction was determined by BCA assay according to manufacturer’s instructions.

### Protein Separation by Two Dimensional-sodium Dodecyl Sulfate-polyacrylamide Gel Electrophoresis (2D-SDS-PAGE)

From the cell lysates, the proteins (500 μg/sample) were first precipitated by five volumes of ice-cold acetone at −28°C for 4 h. After their solubilization in rehydration buffer containing 0.6% dithiothreitol (DTT), passive rehydration of 17 cm nonlinear IPG strips (pH 3–10) was performed for 14 h at RT. The isoelectric focusing (IEF) was carried out using a Bio-Rad Protean IEF cell instrument and the following parameters: (i) 0–150 V in 3 min, (ii) 150 V for 30 min, (iii) 150–300 V in 15 min, (iv) 300 V for 30 min, (v) 300–3500 V in 150 min, (vi) 3500 V for 12 h. For the second dimension, each IPG strip was first incubated for 20 min in equilibration buffer containing 1% DTT and for another 20 min in equilibration buffer containing 2.5% iodoacetamide (IAA). The strips were then placed on a 12% SDS gel and electrophoresis was performed in two steps: the current was first set to 25 mA/gel for ∼30 min and then to 40 mA/gel, until the tracking dye reached the anodic part of the gel. After this separation step, the proteins were prefixed for 1 h with 12% trichloroacetic acid and stained overnight with Coomassie Brillant Blue G-250 containing solution. After destaining the background with 25% methanol in water (v/v), the gels were scanned with a GS-800 calibrated imaging densitometer (Bio-Rad Laboratories GmbH, Munich, Germany) using the QuantityOne software. For each sample, three replicate 2D-gels were comparatively analyzed using the PDQuest 8.0 software (Bio-Rad Laboratories GmbH, Munich, Germany), which allowed automatic detection with manual corrections and quantification of protein spots. The significance of differences between protein spots was evaluated by Student’s t-test and a p value lower than 0.05 was considered as significant. Additional selection criterion was a fold change value higher than two.

### In-gel Tryptic Digestion

Selected protein spots were manually excised and subjected to in-gel tryptic digestion as previously reported [16]. Briefly, destaining of the protein spots was achieved by performing the following steps, which were repeated until the gel pieces were transparent: (i) incubation of the gel pieces with acetonitrile-Milli-Q (3∶2 v/v) solvent mixture for 30 min; (ii) drying using a SpeedVac (Eppendorf AG, Germany) and (iii) rehydration with 20 mM ammonium bicarbonate (pH 8.0) for 15 min. After that, a freshly prepared trypsin solution (12.5 ng/μL of sequencing grade modified trypsin (Promega, Madison, WI, USA) in 20 mM ammonium bicarbonate, pH 8.0) was added to the dried gel pieces and incubated at 4°C for 45 min. Then, the trypsin solution was replaced by 20 mM ammonium bicarbonate (pH 8.0) and incubated at 37°C for 12 h. The tryptic peptides were extracted from the gel with a mixture of acetonitrile-0.1% TFA in Milli-Q water (3∶2, v/v) at RT (3×60 min).

### Mass Spectrometric Analysis and Protein Identification

Tryptic peptide mixtures were analyzed by reversed-phase liquid chromatography-nanospray tandem mass spectrometry (LC-MS/MS) using an LTQ-Orbitrap mass spectrometer (Thermo Fisher Scientific, Bremen, Germany) and an Eksigent nanoHPLC (CA, USA). The characteristics of the reversed-phase LC column were 5 μm, 200 Å pore size C18 resin in a 75 μm i.d.×10 cm long piece of fused silica capillary (Hypersil Gold C18, Thermo Fisher Scientific, Bremen, Germany). After injecting the sample, the column was washed for 5 min with 90% eluent A (0.1% formic acid in water) and 10% eluent B (0.1% formic acid in acetonitrile). The peptides were eluted using a linear gradient of 10–50% eluent B in 25 min and then 50–80% eluent B in 5 min, at a flow rate of 300 nL/min. The LTQ-Orbitrap mass spectrometer was operated in a data-dependent mode in which each full MS scan was followed by five MS/MS scans, where the five most abundant molecular ions were dynamically selected and fragmented by collision-induced dissociation (CID) using a normalized collision energy of 35% in the LTQ ion trap. Dynamic exclusion was allowed. Tandem mass spectra were searched against the SwissProt protein database using Mascot (Matrix Science) with the following parameters: “Trypsin” cleavage with one missed cleavage, cysteine alkylation by iodoacetamide as a constant modification and methionine oxidation as a variable modification.

## Results and Discussion

### Optimization of Cell Treatment Conditions with the Bioconjugate and Free Daunorubicin

The cell treatment conditions (i.e., incubation time and concentration) with the GnRH-III[^4^Lys(Ac), ^8^Lys(Dau = Aoa)] bioconjugate and free Dau were chosen on the basis of *in vitro* cytotoxicity data. The HT-29 human colon cancer cells were treated either with the bioconjugate or free Dau at different concentrations for 6, 24, 48 and 72 h, respectively. Free Dau exerted a cytotoxic effect even after 6 h, which was more pronounced with time. The lowest IC_50_ value (0.26 μM) was determined after 72 h of incubation. In contrast, the bioconjugate was cytotoxic only after 72 h (IC_50_ = 11.5 μM); therefore, the treatment time of 72 h was used in further proteomics studies. The selected cell treatment concentrations were below the IC_50_ values, namely 0.15 μM for free Dau and 3 μM for bioconjugate. It is important to note the different IC_50_ values and consequently different cytotoxic properties of free and conjugated Dau that could be explained by their mechanisms of cellular uptake, namely passive diffusion in the case of free Dau *vs.* receptor-mediated endocytosis, which is followed by intracellular processing of the GnRH-III[^4^Lys(Ac), ^8^Lys(Dau = Aoa)] bioconjugate.

### Changes in the Protein Expression Profile of HT-29 Human Colon Cancer Cells after Chemotherapeutic Treatment

After optimizing the treatment conditions, the HT-29 human colon cancer cells were treated for 72 h with the GnRH-III[^4^Lys(Ac), ^8^Lys(Dau = Aoa)] bioconjugate or free Dau. Cell lysates were prepared according to the protocol described in Materials and Methods section. The protein content of the supernatant fractions was determined by BCA assay and it was in average 2.34 mg/mL for the untreated cells used as a control, 2.94 mg/mL for Dau-treated and 3.18 mg/mL for bioconjugate-treated cells.

**Figure 2:**
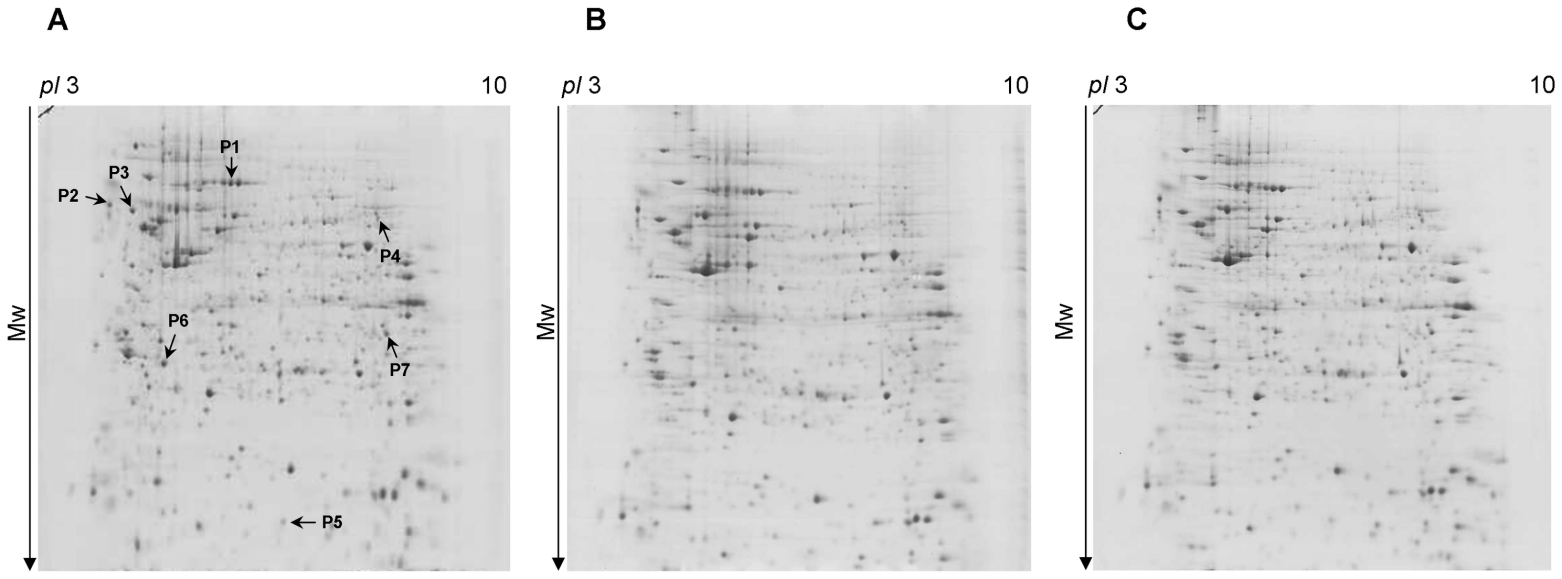
Protein expression profile of HT-29 human colon cancer cells. (A) untreated, (B) bioconjugate-treated and (C) daunorubicin-treated cancer cells. Shown only on the control gel, arrows and spot numbers indicate the significantly different protein spots.

Proteins were then separated by 2D-gel electrophoresis using 3–10 nonlinear IPG strips and 12% gels. After Coomassie staining, the gel patterns were compared using the PDQuest 8.0 software. Differently expressed proteins were subjected to in-gel tryptic digestion, followed by nanoLC-tandem mass spectrometric analysis of the tryptic peptide mixtures and database search. In Figure 2, the analyzed proteins are denoted with an arrow and shown only on the control gel. The proteins found to be differently expressed after targeted chemotherapeutic treatment (fold change >2; p<0.05; Table 1 and Table S1) can be classified in the following functional categories: (i) molecular chaperons, (ii) metabolism-related proteins and (iii) proteins involved in signaling.

**Table 1:** Identified proteins with altered expression due to the chemotherapeutic treatment of HT-29 human colon cancer cells.

Spot Nr.	Identified Protein	Accession Nr.	Mw_calc_ (kDa)	pI_calc_	Control *vs.* Bioconjugatetreated sample		Control *vs.* Dau treatedsample		Bioconjugate *vs.* Dau treatedsample	
					Fold change	p value	Fold change	p value	Fold change	p value
P1	Heat shock 70 kDa protein 1A/1B	P08107	70.0	5.66	3.360	0.0035	1.099	0.4271	0.326	0.0001
P2	Calreticulin	P27797	48.1	4.44	4.340	0.0053	0.860	0.2642	0.198	0.0044
P3	Protein disulfide-isomerase	P07237	57.1	4.87	2.130	0.0049	1.040	0.5561	0.480	0.0007
P4	UDP-glucose 6-dehydrogenase	O60701	55.0	7.12	2.690	0.0250	0.580	0.0040	0.218	0.0051
P5	Fatty acid-binding protein, epidermal	Q01469	15.2	7.01	3.500	0.0059	2.060	0.0177	0.590	0.0322
P6	Ran-specific GTPase-activating protein	P43487	23.3	5.29	2.210	0.05	1.360	0.0637	0.617	0.0010
P7	Guanine nucleotide-binding protein subunit beta-2-like 1	P63244	35.1	7.69	2.250	0.0773	0.910	0.6182	0.400	0.0113

Among molecular chaperones, the expression of heat shock 70 kDa protein 1A/1B (Hsp70) was found to be significantly decreased after treatment with the GnRH-III[^4^Lys(Ac), ^8^Lys(Dau = Aoa)] bioconjugate, while free Dau had no marked effect on its expression in HT-29 human colon cancer cells compared to untreated cells (protein spot P1). Previous reports have indicated that the stress-induced Hsp70 is a molecular chaperone marginally expressed in unstressed normal cells, but overexpressed in different types of cancer cells. Moreover, elevated expression of Hsp70 in cancer cells has been associated with disease progression, metastasis, resistance to chemotherapy and generally poor patient prognosis. These functions may be explained by its anti-apoptotic properties, helping the cells to survive stressful conditions such as the action of chemotherapeutic agents [17]–[19]. The down-regulation of HSP70 has been found to inhibit cell proliferation and induce apoptosis. Thus, Hsp70 has been proposed as an important molecular target in cancer treatment and efforts are being made to develop Hsp70 modulators (e.g., small-molecule inhibitors) [20]–[22]. In our work, the treatment of HT-29 human colon cancer cells with a single dose of free Dau did not affect the expression of Hsp70 protein. Interestingly, the attachment of Dau to a GnRH-III derivative used as a targeting moiety resulted in a bioconjugate with inhibitory properties on Hsp70 (Table 1).

Another protein with chaperone activity, which was found to be differently expressed in HT-29 cells after their treatment with the GnRH-III[^4^Lys(Ac), ^8^Lys(Dau = Aoa)] bioconjugate, was Calreticulin (protein spot P2). The implications of this protein in cancer have recently been reviewed by Zamanian *et al.* [23]. Its overexpression has been reported in different types of cancer cells (e.g., breast, bladder, esophageal, pancreatic, colon, gastric cancer, etc.) and found to be associated with increased invasion, metastasis and poor prognosis [23]. Considering these previous findings, Calreticulin could also represent a cancer therapeutic target, whose decreased expression might provide a therapeutic benefit. In the present study, in contrast to the treatment with free Dau, which had no significant effect on Calreticulin expression, the bioconjugate exerted an inhibitory action (i.e., in comparison with the untreated cells, in the bioconjugate treated ones the level of Calreticulin was in average four times lower; see Table 1).

Compared to untreated and Dau-treated HT-29 colon cancer cells, the treatment with the bioconjugate resulted in decreased expression of protein disulfide isomerase (PDI), a multifunctional protein which plays an important role in protein folding by catalyzing the formation, breaking and rearrangement of intramolecular disulfide bridges (protein spot P3). PDI is known to operate as a chaperone which inhibits the aggregation of incorrectly folded proteins. Furthermore, it has been found that the expression of PDI is elevated in cancer cells and this protein has been proposed as a biomarker for certain types of cancer, such as colon cancer or mammary tumorigenesis [24], [25]. Moreover, Goplen *et al*. have shown that PDI was strongly expressed on invasive glioma cells, playing an important role in tumor cell migration and invasion, actions that could effectively be inhibited by a PDI monoclonal antibody, as well as bacitracin [26].

In addition to molecular chaperones, the expression of metabolism-related proteins such as UDP-glucose 6-dehydrogenase (UGDH) was affected by the chemotherapeutic treatment (protein spot P4). While free Dau had a positive influence on UGDH expression, the application of the bioconjugate had an inhibitory effect. The latter might contribute to cancer therapy, since UGDH antagonists have been proposed as useful therapeutic agents [27], [28]. This is based on the consideration that elevated glycosaminoglycan formation (e.g., hyaluronan), in which UGDH plays a key role, is involved in a variety of human diseases, including tumor progression [29].

Both the bioconjugate and free Dau significantly affected the expression of epidermal fatty acid-binding protein (E-FABP) (protein spot P5). The FABPs are multifunctional proteins involved in lipid metabolism, but also in the modulation of gene expression, growth and survival pathways as well as inflammatory and metabolic responses [30]. Furthermore, altered FABP expression patterns were described for different types of cancer, suggesting that FABPs play an important role in carcinogenesis. For instance, in a recent study by Junqin Li *et al.* it has been found that E-FABP, as well as Liver-FABP and Heart- or Muscle-FABP, are involved in the development of invasive ductal breast cancer, since their levels were significantly elevated in ductal infiltrating carcinoma compared to fibroadenoma [31]. Increased expression of E-FABP was also detected in endometrial cancer and chemoresistant pancreatic cancer cell lines [32], [33]. Thus, E-FABP might represent another molecular target in cancer therapy.

Although not significantly affected by the chemotherapeutic treatment, the expression of Ran-specific GTPase-activating protein (also called Ran-binding protein 1; RanBP1) (protein spot P6) decreased after treatment with the bioconjugate (2.21 fold change, p = 0.05). Ran is a small GTPase that functions as a molecular switch by binding to either GTP or GDP. One essential regulator of this process is Ran-specific GTPase-activating protein (RanBP 1), which also catalyzes the GTP hydrolysis of Ran. It has been found that Ran and Ran binding proteins are involved in a broad range of fundamental cellular processes (e.g., nucleocytoplasmic transport, mitotic spindle assembly, nuclear envelope and nuclear pore complex formation) as well as in cell death, cell proliferation, cell differentiation and malignant transformation (see [34] for review). Moreover, it has been reported that the expression of Ran and RanBP1 is increased in different types of cancer and that the abrogation of RanBP1 may lead to cell death. Taken together these results, it has been suggested that both Ran and Ran-specific GTPase-activating protein are good candidates as molecular targets in cancer therapy [34], [35].

Compared to the untreated HT-29 colon cancer cells, the expression of guanine nucleotide-binding protein subunit beta-2-like 1 (GNB2L1, also known as receptor of activated protein kinase C 1, RACK1) (protein spot P7) was influenced by the treatment with the bioconjugate, but not significantly (2.25 fold change; p = 0.0773). However, the expression of this protein was significantly different in the bioconjugate *vs.* Dau treated cells (p = 0.0113), a lower amount being detected in the bioconjugate treated cells. GNB2L1 has been found to play a crucial role in multiple intracellular signal transduction pathways. Regarding its possible implications in cancer development and progression, it has been shown that RACK1 promotes breast carcinoma proliferation and invasion/metastasis *in vitro* and *in vivo* and its expression is associated with poor prognosis. Furthermore, reduction of RACK1 expression led to the inhibition of cell proliferation *in vitro* [36]. Similar results have also been reported in case of other types of cancer such as non-small cell lung and colon carcinoma [37].

In conclusion, the treatment with the daunorubicin-GnRH-III derivative bioconjugate resulted in changes in the protein expression profile of HT-29 colon cancer cells. In particular, molecular chaperons, metabolism-related proteins and proteins involved in signaling were affected by the targeted chemotherapeutic treatment, their expression being down-regulated in the bioconjugate treated cells compared to the untreated and Dau-treated ones. Previous studies have demonstrated the implications of these proteins in cancer, indicating that their down-regulation might be of therapeutic benefit (e.g., Hsp70, protein disulfide isomerase, etc). Recent progress in cancer therapy has suggested the importance of targeting more than one protein or signaling pathway. One possible approach to achieve this could be targeted cancer chemotherapy. On the basis of our results, it can be concluded that the GnRH-III[^4^Lys(Ac), ^8^Lys(Dau = Aoa)] bioconjugate exerts its cytotoxic action on HT-29 colon cancer cells by interfering with multiple intracellular processes and represents a promising targeted chemotherapeutic agent.

## Supporting Information

Figure S1 **Synthesis of oxime bond-linked daunorubicin-GnRH-III derivative bioconjugate.**
(TIF)

Figure S2 **Chemical characterization of GnRH-III[^4^Lys(Ac), ^8^Lys(Dau = Aoa)] bioconjugate.** (A) analytical HPLC profile and (B) ESI-ion trap mass spectrum. Fragmentation of glycosidic bonds under mass spectrometric conditions, leading to the loss of daunosamine, is denoted by an asterisk.
(TIF)

Table S1 **Characteristics of the identified proteins with altered expression due to the chemotherapeutic treatment of HT-29 colon cancer cells.**
(DOC)

Protocol S1 **Synthesis of aminooxyacetylated-GnRH-III derivative.**
(DOC)

Protocol S2 **High performance liquid chromatography (HPLC).**
(DOC)

Protocol S3 **Mass spectrometric analysis.**
(DOC)
