# Supplementary figures and images for: Protein Expression Profile of HT-29 Human Colon Cancer Cells after Treatment with a Cytotoxic Daunorubicin-GnRH-III Derivative Bioconjugate
Source: PLoS One. 2014 Apr 9;9(4):e94041. doi: 10.1371/journal.pone.0094041 (PMC3981732; doi:10.1371/journal.pone.0094041)

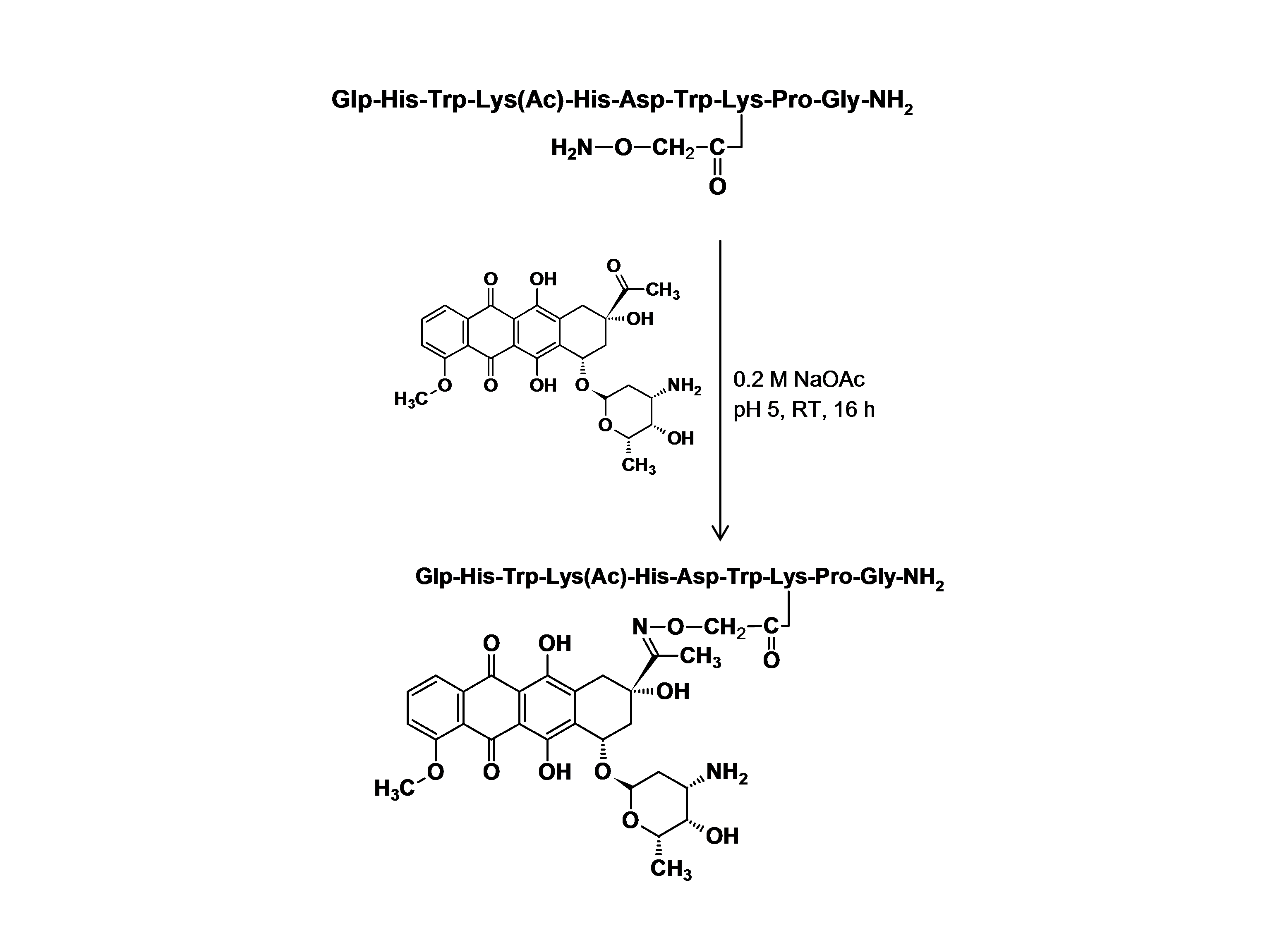

Supplement: Figure S1 — Synthesis of oxime bond-linked daunorubicin-GnRH-III derivative bioconjugate. (TIF) [file pone.0094041.s001.tif]

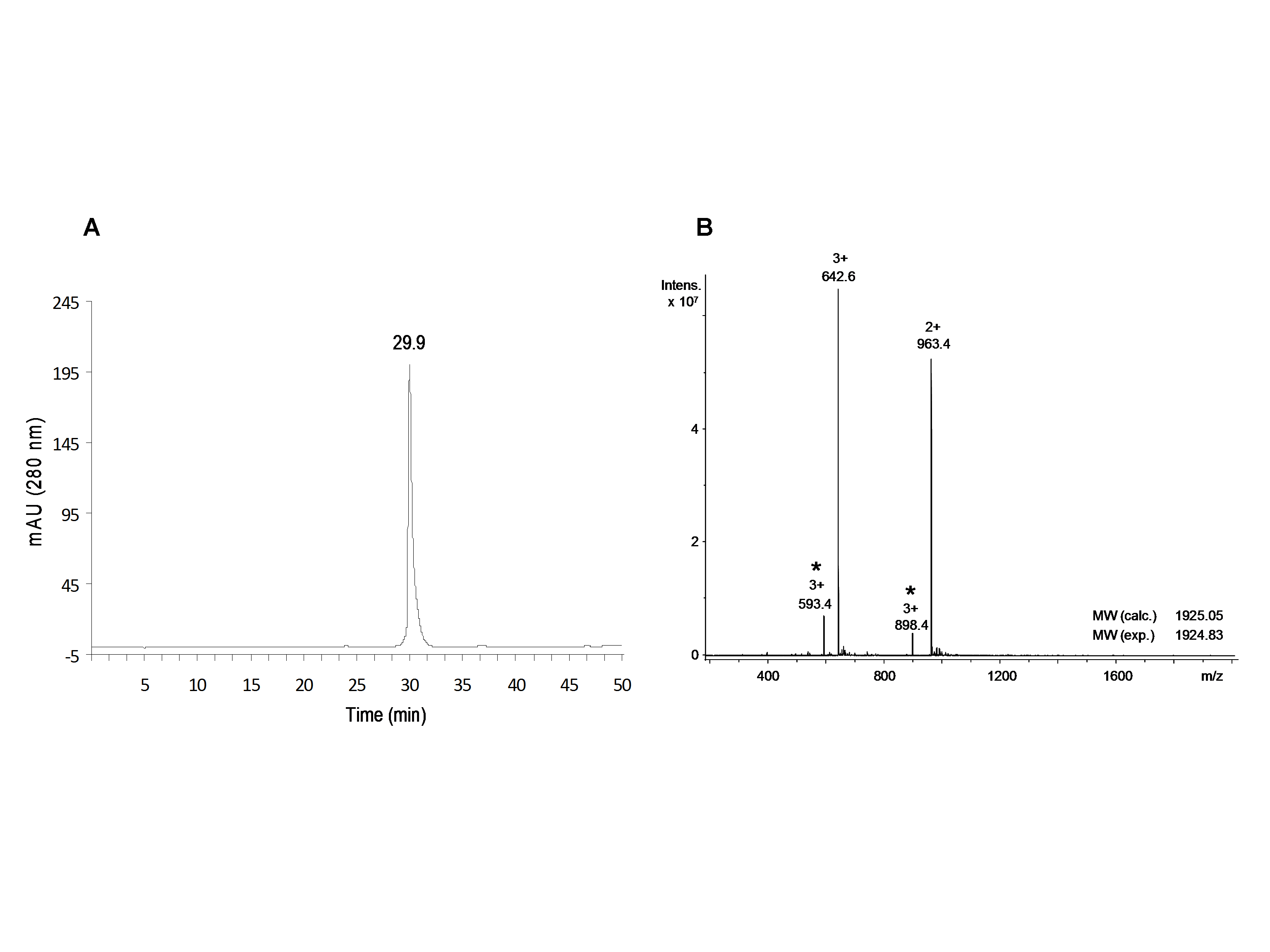

Supplement: Figure S2 — Chemical characterization of GnRH-III[4Lys(Ac), 8Lys(Dau = Aoa)] bioconjugate. (A) analytical HPLC profile and (B) ESI-ion trap mass spectrum. Fragmentation of glycosidic bonds under mass spectrometric conditions, leading to the loss of daunosamine, is denoted by an asterisk. (TIF) [file pone.0094041.s002.tif]
