# Supplementary material for: Protein Expression Profile of HT-29 Human Colon Cancer Cells after Treatment with a Cytotoxic Daunorubicin-GnRH-III Derivative Bioconjugate
Source: PLoS One. 2014 Apr 9;9(4):e94041. doi: 10.1371/journal.pone.0094041 (PMC3981732; doi:10.1371/journal.pone.0094041)
Supplement: Table S1 — Characteristics of the identified proteins with altered expression due to the chemotherapeutic treatment of HT-29 colon cancer cells. (DOC) [file pone.0094041.s003.doc]

**Table S1.** Characteristics of the identified proteins with altered expression due to the chemotherapeutic treatment of HT-29 colon cancer cells

| **Spot Nr.** | **Identified Protein** | **Accessio Nr.** | **Mw_calc_ (kDa)** | **pI_calc_** | **Coverage** | **# PSMs** | **# Peptides** | **# AAs** | **Score** |
| --- | --- | --- | --- | --- | --- | --- | --- | --- | --- |
| P1 | Heat shock 70 kDa protein 1A/1B | P08107 | 70.0 | 5.66 | 18.25 | 7 | 7 | 641 | 394.47 |
| P2 | Calreticulin | P27797 | 48.1 | 4.44 | 46.04 | 60 | 13 | 417 | 2458.73 |
| P3 | Protein disulfide-isomerase | P07237 | 57.1 | 4.87 | 49.80 | 67 | 20 | 508 | 1616.29 |
| P4 | UDP-glucose 6-dehydrogenase | O60701 | 55.0 | 7.12 | 68.42 | 57 | 24 | 494 | 1995.93 |
| P5 | Fatty acid-binding protein, epidermal | Q01469 | 15.2 | 7.01 | 57.78 | 39 | 9 | 135 | 980.57 |
| P6 | Ran-specific GTPase-activating protein | P43487 | 23.3 | 5.29 | 10.95 | 6 | 2 | 201 | 209.77 |
| P7 | Guanine nucleotide-binding protein subunit beta-2-like 1 | P63244 | 35.1 | 7.69 | 53.31 | 16 | 12 | 317 | 487.74 |
