# Supplementary material for: Protein Expression Profile of HT-29 Human Colon Cancer Cells after Treatment with a Cytotoxic Daunorubicin-GnRH-III Derivative Bioconjugate
Source: PLoS One. 2014 Apr 9;9(4):e94041. doi: 10.1371/journal.pone.0094041 (PMC3981732; doi:10.1371/journal.pone.0094041)
Supplement: Protocol S1 — Synthesis of aminooxyacetylated-GnRH-III derivative. (DOC) [file pone.0094041.s004.doc]

**Protocol S1.** **Synthesis of aminooxyacetylated-GnRH-III derivative**

The aminooxyacetylated (Aoa) derivative of GnRH-III (<EHWK(Ac)HDWK(Aoa)PG-NH_2_, where <E is pyroglutamic acid) was prepared manually by solid phase peptide synthesis according to Fmoc/tBu chemistry on a Rink-Amide MBHA resin (0.38 mmol/g coupling capacity). The following Fmoc-protected amino acid derivatives were used: Fmoc-Gly-OH, Fmoc-Pro-OH, Fmoc-Lys(Mtt)-OH, Fmoc-Lys(Dde)-OH, Fmoc-Trp(Boc)-OH, Fmoc-Asp(OtBu)-OH and Fmoc-His(Trt)-OH.

The protocol of the synthesis was as follows: (i) DMF washing (4 x 1 min), (ii) Fmoc deprotection with 2% DBU, 2% piperidine in DMF (2 + 2 + 5 + 10 min), (iii) DMF washing (10 x 1 min), (iv) coupling of 5 equiv α-Fmoc-protected amino acid/PyBOP/NMM(1:1:2) in DMF (60 min), (v) DMF washing (4 x 1 min). After completion of the synthesis of the protected decapeptide, the Dde-protecting group of the ε-NH_2_ group of ^4^Lys was removed by 2% hydrazine in DMF (2 x 15 min); subsequently, the acetylation of the free ε-NH_2_ group was achieved by treatment with Ac_2_O/DIPEA/DMF (1:1:3, v/v/v) for 1 h. Thereafter, the Mtt-protecting group of ^8^Lys was removed with 2% TFA in DCM (6 x 5 min) and then bis-Boc-Aoa-OH was attached to the free ε-NH_2_ group of ^8^Lys after preactivation with PyBOP in the presence of NMM (5 and 10 equiv to the resin capacity, respectively; coupling time 45 min). The aminooxyacetylated peptide was cleaved from the resin using a mixture of 95% TFA, 2.5% TIS and 2.5% water (v/v/v) for 2.5 h at room temperature and then precipitated with ice-cold diethyl ether, washed three times with diethyl ether and solubilized in 100% acetic acid prior to freeze drying. The crude product was purified by semipreparative RP-HPLC and analyzed by mass spectrometry.
