# Supplementary material for: Protein Expression Profile of HT-29 Human Colon Cancer Cells after Treatment with a Cytotoxic Daunorubicin-GnRH-III Derivative Bioconjugate
Source: PLoS One. 2014 Apr 9;9(4):e94041. doi: 10.1371/journal.pone.0094041 (PMC3981732; doi:10.1371/journal.pone.0094041)
Supplement: Protocol S2 — High performance liquid chromatography (HPLC). (DOC) [file pone.0094041.s005.doc]

**Protocol S2. High performance liquid chromatography (HPLC)**

The crude product (GnRH-III[^4^Lys(Ac),^8^Lys(Dau=Aoa)] bioconjugate) was purified on an UltiMate 3000 HPLC system (Dionex, Idstein, Germany) using a semipreparative Vydac C18 column (250 mm x 10 mm) with 10 μm silica (300 Å pore size). Linear gradient elution (0 min 20% B; 5 min 20% B; 55 min 70% B) with eluent A (0.1% TFA in water) and eluent B (0.1% TFA in MeCN-H_2_O (80:20, v/v)) was used at a flow rate of 4 mL/min. Peaks were detected at 220 and 280 nm.

Analytical RP-HPLC was performed on an UltiMate 3000 system (Dionex, Idstein, Germany) using a Vydac C18 column (250 mm x 4.6 mm) with 5 μm silica (300Å pore size) as a stationary phase. Linear gradient elution (0 min 0% B; 5 min 0% B; 50 min 90% B) with eluent A (0.1% TFA in water) and eluent B (0.1% TFA in MeCN-H_2_O (80:20, v/v)) was used at a flow rate of 1 mL/min. Peaks were detected at 280 nm.
