# Supplementary material for: Protein Expression Profile of HT-29 Human Colon Cancer Cells after Treatment with a Cytotoxic Daunorubicin-GnRH-III Derivative Bioconjugate
Source: PLoS One. 2014 Apr 9;9(4):e94041. doi: 10.1371/journal.pone.0094041 (PMC3981732; doi:10.1371/journal.pone.0094041)
Supplement: Protocol S3 — Mass spectrometric analysis. (DOC) [file pone.0094041.s006.doc]

## Protocol S3. Mass spectrometric analysis

Electrospray (ESI)-mass spectrometric analysis of GnRH-III[^4^Lys(Ac),^8^Lys(Dau=Aoa)] bioconjugate was carried out on an Esquire 3000+ ion trap mass spectrometer (Bruker Daltonics, Bremen, Germany). Spectra were acquired in the 50 - 2500 *m/z* range. The sample was dissolved in a mixture of 50% methanol, 48% water and 2% acetic acid.
